# Assessment of the Impact of Clothing Structures for Premature Babies on Biophysical Properties

**DOI:** 10.3390/ma14154229

**Published:** 2021-07-29

**Authors:** Ewa Skrzetuska, Adam K. Puszkarz, Zofia Pycio, Izabella Krucińska

**Affiliations:** Institute of Material Science of Textiles and Polymer Composites, Faculty of Material Technologies and Textile Design, Lodz University of Technology, 116 Żeromskiego Street, 90-924 Lodz, Poland; ewa.skrzetuska@p.lodz.pl (E.S.); z.pycio@gmail.com (Z.P.); izabella.krucinska@p.lodz.pl (I.K.)

**Keywords:** biophysical comfort, thermal insulation, heat transfer, mass transfer, protective clothing, knitted fabric, newborns, CAD modeling, simulation, micro-CT

## Abstract

This article presents research on ergonomics and physiological comfort of protective clothing. Biophysical properties of selected three-layer textile assemblies that differ in geometry and raw material composition for the production of types of mummy sleeping bags for premature babies were investigated. The tests included measurements of air permeability, thermal resistance and water vapor resistance (both by means of human skin model), thermal insulation, and water vapor resistance (both using newborn manikin). Experimental research was supplemented by modeling the thermal insulation of the assemblies by designing their 3D models using selected CAD software and applying the finite volume method. The obtained results allowed the evaluation of the influence of different geometry and the raw material composition of the proposed assemblies on the performance parameters of protective clothing.

## 1. Introduction

Preterm babies belong to a group of users with special-purpose clothing with specific biophysical properties resulting from the immature structure of the skin and immaturity for its proper functioning [1]. According to the World Health Organization definition, each infant born before 37 weeks of gestation is included in the group of premature babies. There is also a classification of the degree of prematurity in terms of childbirth in a specific week of pregnancy: extremely premature labor (less than 28 week), very premature labor (28–31 weeks), moderately premature labor (32–33 weeks), and moderately premature labor (34–36 weeks). Preterm babies are also distinguished by body weight: low body weight (1500–2499 g), very low body weight (1000–1499 g), and extremely low body weight (500–999 g) [2].

According to world statistics, the number of premature births is constantly increasing, which, referring to the reports by American researchers, results from the increase in the number of newborns born between 34–36 weeks of pregnancy. They constitute as much as 70% of all premature babies [3].

Premature births result from low awareness of future mothers, environmental pollution, poverty, or genetic conditions. In the perinatal period, there is an increased risk of infections, causing malformations and diseases that may result in perinatal mortality, i.e., the number of stillbirths weighing at least 1000 g and deaths in the first week after delivery. According to the reports of Statistics Poland, the perinatal mortality in 2015 decreased to 4.43 per mille, compared to 4.96 per mille a year earlier. This indicator in Poland is decreasing year by year due to the development of perinatal care, reference levels of standards, perinatal care, and a new specialization program in obstetrics and gynecology that was implemented in 2010. However, the mortality rate of premature children in Poland is still higher than in other countries Western Europe [4,5,6].

The skin, which is the largest human organ, is responsible for creating a barrier between the body and the environment, thus protecting the body against any mechanical damage or bacteria and infections. In addition, biological stimuli and microbes play an important role in regulating fluids and temperature and also mediate the senses of heat, cold, touch, pain, and comfort. The subcutaneous tissue of premature babies consists of small fat cells that are composed of white fat, which provides insulating functions and is a source of energy, and brown fat, which plays an important role in thermoregulation processes. Only in the 26th week of pregnancy does the accumulation of subcutaneous fat in the fetus begin. The amount of white fat increases as pregnancy progresses, while brown tissue is negligible or is often completely absent. This tissue is a deeper structure that generates heat through fatty acid oxidation. For this reason, premature babies lose heat more intensively than full-term babies [1].

Newborn babies dissipate heat through four mechanisms: evaporation, conduction, convection, and radiation, where evaporation of sweat causes heat loss, while convection, radiation and conduction can both contribute to heat loss and recovery. The immaturity of the skin of premature babies, including the lack of the stratum corneum, contributes to the loss of heat and the reduction of body moisture. The earlier the week that a premature baby is born in, the more transparent, thin and gelatinous, and red-colored his skin is, and its thickness can reach up to 27.4 µm [7].

At birth, a premature baby leaves a warm and humid environment to a cold and dry environment and has a large body surface area in relation to its body weight, resulting in the loss of water and heat through evaporation. It has been found that in the first 24 h of life in premature babies with a low birth weight, water loss is 150 mL per 1 kg of body weight, which can lead to electrolyte imbalance. In addition, increased movement or crying after childbirth increases the risk of hypothermia. To prevent this, the newborn is temporarily placed in a polyethylene bag, which reduces the loss of heat and moisture by creating a microclimate around the baby’s body. There are also other alternative methods of protecting a premature baby, such as the use of creams, ointments, or occlusive bio-dressings. Currently, incubators in which the humidity and temperature of the environment are regulated are the most effective, but even then, there is a risk of harmful pathogens developing. Moreover, the prevailing humidity in the incubator is not conducive to the skin’s production of a protective barrier, which in turn delays the skin maturation process of a premature baby. For this reason, the initially maintained humidity at the level of approx. 80% in incubators, is reduced by 0.5–10% daily to normalize it to the level of approx. 40% after the premature baby reaches 32 weeks of age. The rate of changes in the humidity level in the incubator depends on the week of delivery and the body weight of the newborn, while the environmental temperature must be constantly kept within the narrow limits of 36.5–37.5 °C to minimize oxygen consumption and caloric consumption by the body. It is conducive to the development of the premature baby’s body weight and helps to maintain heat balance [1,7].

Preterm babies must be monitored through a series of tests and procedures, during which they are usually taken out of the incubator. The baby should be dressed in advance to avoid hypothermia. Due to the specificity of the skin of premature babies, clothes must meet many requirements. The existing solutions include mainly cotton clothes, because this material is natural, delicate, and airy, and it is thanks to this that it does not irritate the premature baby’s skin and provides sensory comfort. However, when a premature baby loses moisture, cotton absorbs the excess, and by contact with the skin, it can cool the baby’s body, causing thermal discomfort. Moreover, wet clothing lowers the thermal insulation properties of the knitted fabric, thus increasing the heat transfer coefficient [1].

Other solutions are constantly being sought that would fully ensure biophysical comfort by adequately keeping in heat while removing the moisture from the skin into the environment without accumulating it excessively in the material at the same time. Polymer materials have a lower thermal conductivity than cotton, and it is because of this that they do a better job at retaining body heat or moisture, but unfortunately, they are artificial materials, so it is difficult to drain moisture into the environment and create proper air exchange between the skin and the environment. Solutions in the form of systems of these mentioned materials are considered in order to simultaneously achieve all of the criteria for appropriate conditions of biophysical comfort for the demanding skin and the organism of a premature baby. Tests are conducted with various thicknesses of cotton as well as with various artificial materials, such as vapor permeable membranes or polymer knitted fabrics considered in different configurations. Of course, the devices on which the research is conducted belong to theoretical models, because the functioning of the natural organism is not a repeatable process, even under the same conditions for the same person. Parameters that can be determined and allow us to come closer to the ideal solution are air permeability, thermal resistance and water vapor resistance of material systems as well as effective thermal insulation and effective water vapor resistance of the manufactured clothes.

The aim of this study was to make a clothing structure in the form of a sleeping bag and cap from six three-layer textile assemblies to conduct biophysical tests in accordance with the applicable standards. It was important to compare the simulated tests with the use of a skin model and an innovative thermal manikin of a premature child to check whether the developed three-layer systems has better thermal insulation and water vapor resistance than the currently used solutions.

Currently, numerical modeling is an effective and widely used tool to investigate thermal and mechanical processes occurring in textiles, among other things [8,9,10,11,12,13,14,15,16,17,18,19,20,21,22,23,24,25,26,27]. In the current work, the experimental research was supplemented by the simulation of the thermal insulation of the assemblies by designing their 3D models using selected CAD software and applying the finite volume method. The simulation portion of the current work is a continuation of the research on numerical modeling of physical phenomena occurring in protective clothing that has previously been presented [28,29,30,31,32,33,34].

## 2. Materials and Methods

### 2.1. Materials

**Figure 1:**
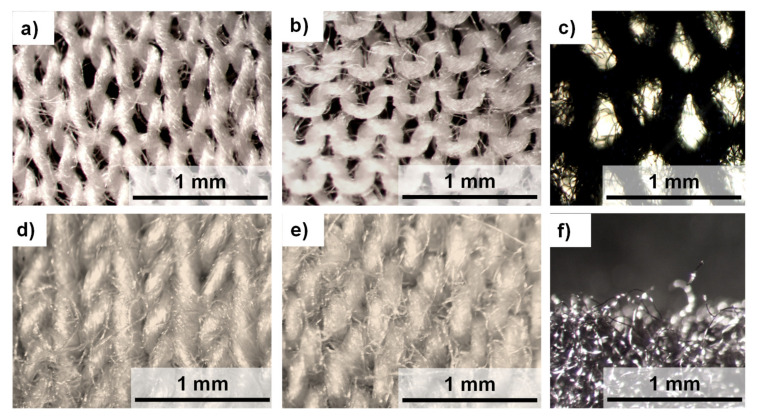
Optical microscopy images of knitted fabric made of Cotton LM ((**a**)—right side, (**b**)—left side), knitted fabric made of Cotton HM ((**c**)—right side, (**d**)—left side), brushed knitted fabric made of PES ((**e**)—right side, (**f**)—brushed part (side view)).

The subject of the research was six three-layer textile assemblies (*A*–*F*) of different compositions. The individual layers were the following textiles: knitted fabric made with Cotton LM (knitted fabric of low mass per unit area), knitted fabric made with Cotton HM (knitted fabric of higher mass per unit area), brushed knitted fabric made with polyester (PES), vapor permeable membrane made with polyurethane (PU), and vapor permeable membrane made with polypropylene (PP). Parameters of the assemblies are presented in Table 1. Optical images of the knitted fabrics are presented in Figure 1.

The construction of knitted fabrics intended for clothing for newborns should be varied depending on the development phase of the organism. In the case of premature newborns, a knitted construction should be used with properties blocking the moisture flow and should be characterized by a high thermal insulation, while ensuring adequate comfort of use. Providing knitted products with the above properties can be achieved through a three-layer construction and the use of raw materials with a different affinity for moisture. In the proposed assemblies, we can distinguish two knitted fabrics with high biophysical values and a vapor-permeable membrane that limits the release of moisture from the body of a premature baby. For this purpose, the layer adhering directly to the skin made of a knitted fabric with conductive-diffusion properties was selected, the role of which is to drain and transport moisture from the body, both in the form of a liquid and gas phase. The outer layer is a knitted fabric with sorption properties, the role of which is to keep moisture away from the premature newborn’s body and evaporate it into the environment.

Knitted fabrics were selected for the production of sleeping bags for babies because they have better sensory properties, are softer, and are more delicate than woven or non-woven products. Moreover, the materials for clothing for premature babies should be friendly to the sensitive skin of the newborn and have the lowest possible surface mass in order not to load the delicate body of the premature baby. In order to develop sleeping bags ensuring high biophysical comfort, materials have been selected that are characterized by a low surface mass and thickness. Analyzing the results summarized in Table 1 for materials constituting individual layers, the polyester brushed knitted fabric of 106.3 g·m^−2^ has the highest area weight, and the polyurethane vapor permeable membrane of 14.8 g·m^−2^ has the lowest weight. Thus, for three-layer assemblies, the highest results of the tested parameters were observed for Assembly *E* (244.7 g·m^−2^), while the lowest results were obtained for the assembly containing two layers of low-mass cotton (LM) and polyurethane vapor permeable membrane—Assembly *C* (158.8 g·m^−2^). The polyester brushed knitted fabric has the highest thickness (0.558 mm), and the polyurethane vapor permeable membrane has the lowest thickness (0.013 mm). The thickest, Assembly *E* (1.942 mm), consists of knitted cotton with a standard mass per unit area (HM), a polypropylene vapor permeable membrane, and a polyester brushed knitted fabric, while the lowest thickness, Assembly *C* (0.788 mm), consists of two layers of knitted cotton with a low mass per unit area and a polyurethane vapor permeable membrane.

**Figure 2:**
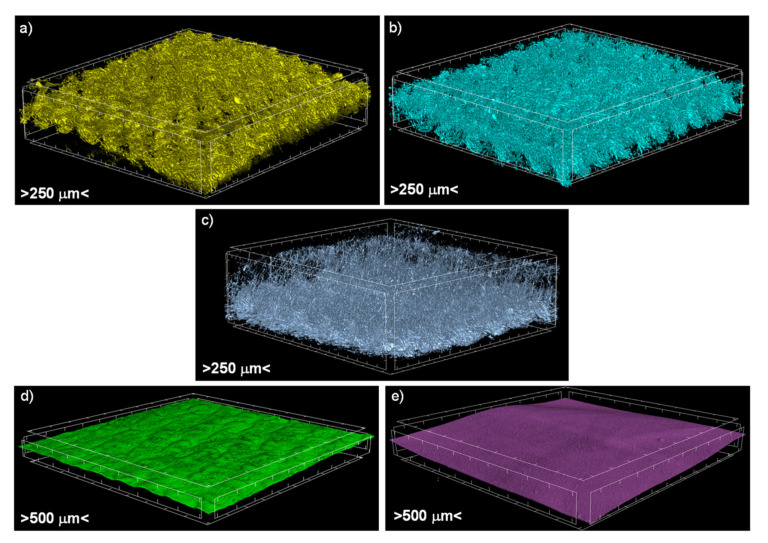
Micro-CT images of (**a**) knitted fabric made of Cotton LM, (**b**) knitted fabric made of Cotton HM, (**c**) brushed knitted fabric made of PES, (**d**) vapor permeable membrane made of PU, (**e**) vapor permeable membrane made of PP.

**Figure 3:**
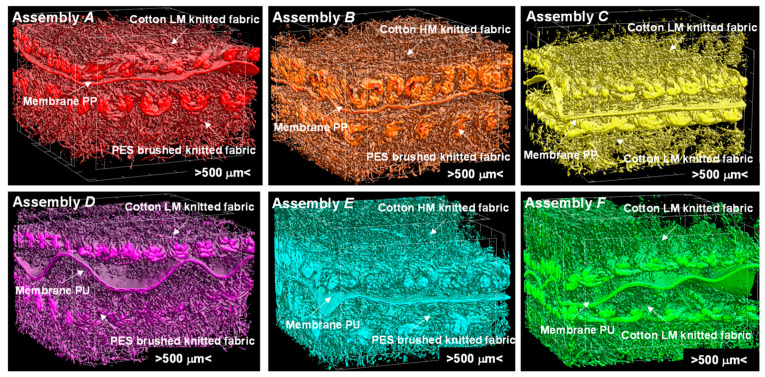
Micro-CT images of tested assemblies.

A 3D reconstruction of all of the textiles constituting the six tested assemblies made using X-ray micro-computed tomography (SkyScan 1272; Bruker, Kontich, Belgium) are presented in Figure 2. Additionally, reconstruction of all of the assemblies are presented in Figure 3. Micro-CT images were obtained by applying the following scanning conditions: X-ray source voltage 50 kV, X-ray source current 200 µA, and pixel size 4.5 µm. A 180° rotation was performed with a rotation step of 0.2°, and no filter was selected.

### 2.2. Methods

#### 2.2.1. Experiment

One of the known models for assessing the biophysical properties of protective clothing and is based on studies conducted in a fixed heat flow is the Umbach method. It is a five-step model describing the course of research on the biophysical properties of clothing in the production and utility processes [7].

The first step focuses on the appropriate selection of materials and testing their properties under stationary conditions on a skin model that simulates the release of heat and moisture through human skin to the external environment through clothing layers. The second stage focuses on the assessment of the biophysical properties of the designed clothing, tested using tests conducted on a thermal manikin that maintains a constant temperature close to the temperature of the human body, and in our case, a model imitating the figure of a prematurely born child weighing about 1500 g. In the case of the tests conducted in this work, the manikin was stationary and lying down. Only these first two steps focus on studying the biophysical properties of the garment under steady heat flux conditions [7].

##### Thermal and Vapor Resistance (Human Skin Model)

**Figure 4:**
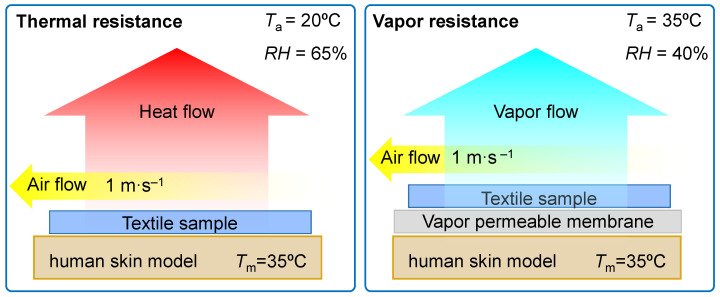
Conditions of the textile thermal and vapor resistance measurements.

The purpose of this experimental part was to evaluate the thermal and water vapor resistance using a measuring stand constructed by Measurement Technology Northwest called Sweating Hotplate 8.2 according to PN-EN ISO 11092:2014-11 [33], ISO 7243 [34], and [7,35]. The parameter related to the evaluation of the thermal resistance is calculated according to the following formula:

(1) $$R_{ct}=\frac{\left(T_{m}-T_{a}\right)\cdot A}{H-H_{c}}-R_{ct0}$$

where *T*_m_ is the heating plate temperature (°C), *T*_a_ is the air temperature (°C), *A* is the surface of the measuring plate (m^2^), *H* is the heating power supplied to the measuring plate (W), ∆*H*_c_ is the heating power correction in case of measuring thermal resistance (W), and *R_ct_*_0_ is the instrument constant for measuring thermal resistance, (m^2^·°C·W^−1^), and the parameter related to the assessment of water vapor resistance *R*_et0_ (m^2^·Pa·W^−1^) is calculated according to the formula:

(2) $$R_{et}=\frac{\left(p_{m}-p_{a}\right)\cdot A}{H-H_{e}}-R_{et0}$$

where *p*_m_ is the saturated water vapor partial pressure (Pa) at the surface of the measuring plate at the temperature *T*_m_, *p*_a_ is the partial pressure (Pa) of water vapor in the air in the measuring chamber at the temperature *T*_a_, *A* is the surface of the measuring plate (m^2^), *H* is the heating power (W) supplied to the measuring plate, ∆*H*_e_ is the heating power correction (W) in the case of measuring the water vapor resistance, and *R_et_*_0_ is the instrument constant (m^2^·Pa·W^−1^) for the measurement of water vapor resistance according to ISO 7243 [34] and [7]. The tests were carried out consecutively under the following conditions: thermal resistance—*T*_a_ = 20 °C, *RH* = 65%, air flow speed 1 m·s^−1^ and water vapor resistance—*T*_a_ = 35 °C, *RH* = 40%, air flow speed 1 m·s^−1^ (Figure 4)

##### Thermal Insulation and Water Vapor Resistance (Newborn Manikin)

**Figure 5:**
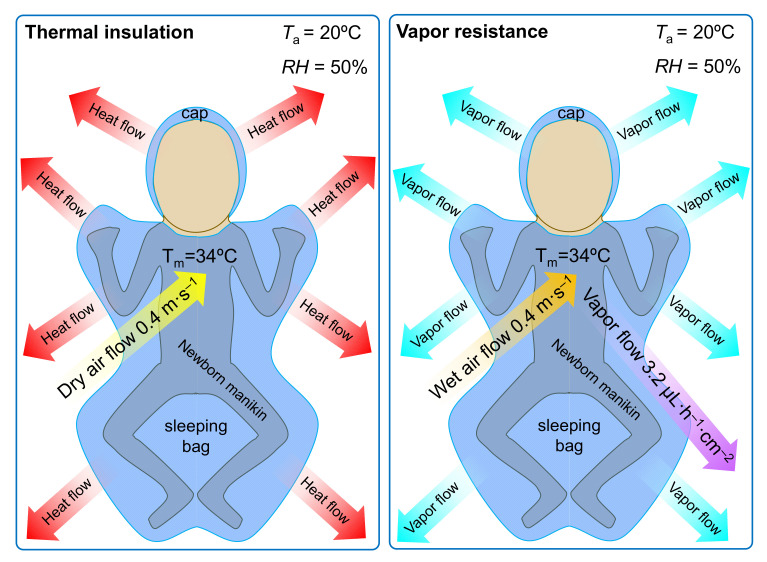
Manikin conditions for thermal insulation and vapor resistance clothing measurements.

As a part of Umbach’s second-degree analysis, the assessment of thermal insulation and water vapor resistance was carried out using an innovative measuring stand, i.e., a premature baby manikin made of 11 heating zones [36]. This model corresponds to the size and proportions of the body of a prematurely born child with a birth weight of approximately 1500 g. Its dimensions were based on the actual dimensions of the premature babies in the hospital of the Children’s Memorial Health Institute in the Neonatology Department in 2012. A full description of the premature baby thermal mannequin with a function of moisture secretion was presented in earlier article [36]. The thermal manikin allows for the determination of the biophysical properties of the tested clothing by calculating its thermal insulation and water vapor resistance in conditions presented in Figure 5. According to PN-EN ISO 15831:2006 [37], thermal insulation is the temperature difference between the user’s skin surface and the surrounding atmosphere divided by the dry heat flux per unit area measured in the direction of temperature gradient. The dry heat flux includes the flux transported by conduction, convection, and heat radiation and is determined by measuring the power of the current supplied to the individual heating zones of the manikin in order to maintain a constant temperature gradient between the manikin’s surface and the surrounding air, and it is related to the area of the entire manikin [7,35,36]. The total thermal insulation of the clothing is expressed in m^2^·°C·W^−1^.

Another parameter calculated by the thermal manikin method is the water vapor resistance expressed in m^2^·Pa·W^−1^. It determines the ratio of the water vapor pressure difference at the infant’s skin and the environment and the loss of the vapor heat flux resulting from water evaporation, during which the pressure equalizes on both sides of the garment [7,35,36]. The tests with dry and wet heat flux were conducted at the air flow speed of 0.4 m·s^−1^, an ambient temperature of 20 °C, an air humidity of 50%, and the temperature of the preterm manikin at 34 °C. Additionally, during the test in the wet heat flux conditions, the moisture expenditure was 3.2·µL·h^−1^·cm^−2^.

##### Air Permeability

**Figure 6:**
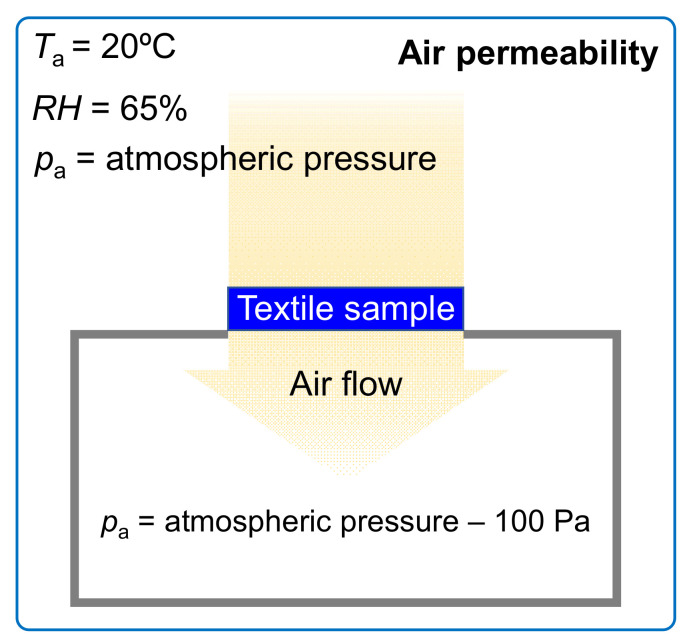
Conditions for textile air permeability measurements.

The air permeability measurements of the tested assemblies were conducted according to PN-EN ISO 9237:1998 [38] in normal climate: relative humidity—*RH* = 65%, air temperature—*T*_a_ = 20 °C, and air pressure—*p*_a_ = 1013.25 hPa, (Figure 6) by means of an air permeability tester (FX 3300 model made by Textest Instruments in Schwerzenbach, Switzerland).

As a result of the constant pressure difference (maintained by the tester), air flow through the tested assembly perpendicular to its surface occurred. The device measures air flow leaving the textile. The air permeability of each assembly was determined as the average of 10 independent measurements at a pressure difference of 100 Pa. The surface area of the sample was 20 cm^2^.

#### 2.2.2. Modeling

##### Model Designing

**Figure 7:**
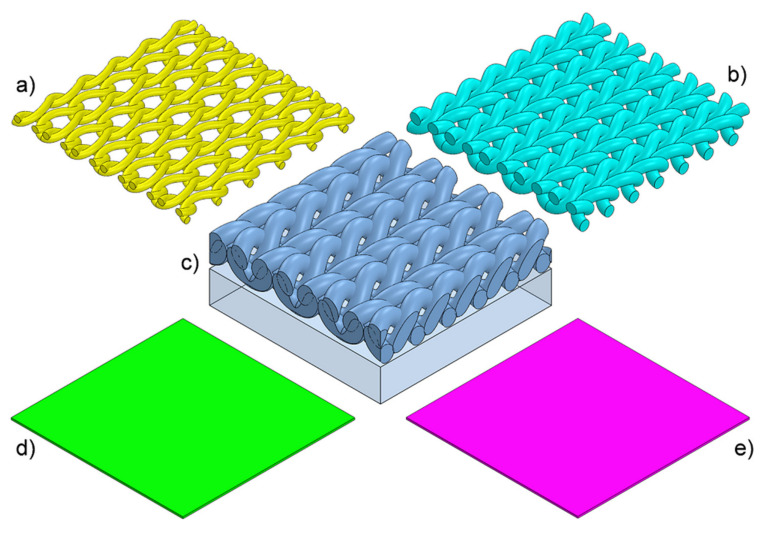
Images of 3D models of (**a**) knitted fabric made of Cotton LM, (**b**) knitted fabric made of Cotton HM, (**c**) brushed knitted fabric made of PES, (**d**) vapor permeable membrane made of PU, and (**e**) vapor permeable membrane made of PP.

The three-dimensional geometric models of six tested assemblies were designed using Solidworks 2014 CAD software [39]. First, 3D models of all five textiles making up layers of the assemblies were designed: (1) knitted fabric made of Cotton LM, (2) knitted fabric made of Cotton HM, (3) brushed knitted fabric made of PES (built of loops layer and brushed layer), (4) a vapor permeable membrane made of PU, and (5) a vapor permeable membrane made of PP (Figure 7).

**Figure 8:**
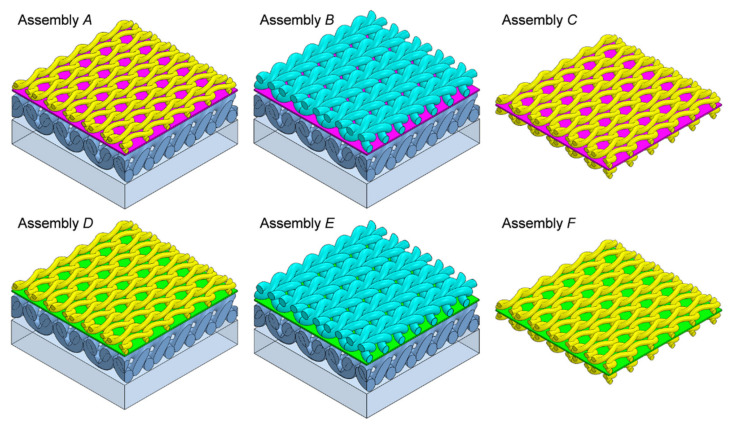
Images of 3D models of six tested assemblies.

The following geometric parameters were taken into account in the models of all three knitted fabrics: (1) layer thickness, (2) loop shape (3) distance between loops, and (4) elliptic cross-section of yarns. The yarn in all of the knitted fabrics models was designed as a monofilament (without taking the individual fibers and the spaces between them filled with air into account). The three knitted fabric models and the two vapor permeable membrane models were then used to design the 3D models of the six tested assemblies (Figure 8). In the designed models, the layers lie on top of each other in direct contact. The models of the tested assemblies do not take individual threads connecting layers into account due to their negligibly low weight and negligibly low influence on heat transfer inside the assembly.

##### Simulations

- Physical basis.

Heat transfer simulations inside 3D models of tested textile assemblies were performed using finite volume method by Solidworks Flow Simulation 2014 software). This computational method allows the prediction of the fluid flow solving energy conversation and Navier–Stokes equations [40]. The above-mentioned equations are extended by equations of fluid state and by the dependence of fluid density, viscosity, and thermal conductivity on fluid temperature. The applied method can be analyzed by following physical phenomena: (1) heat transfer in solids (conduction); (2) free, forced, and mixed convection; and (3) radiation, both in the steady state and transient state [40]. A fuller description of the physical basis of which the modeling was conducted was shown in an earlier work on the modeling of the thermal performance of multilayer protective clothing that is exposed to radiant heat [29].

- Conditions of heat transfer simulations.

**Figure 9:**
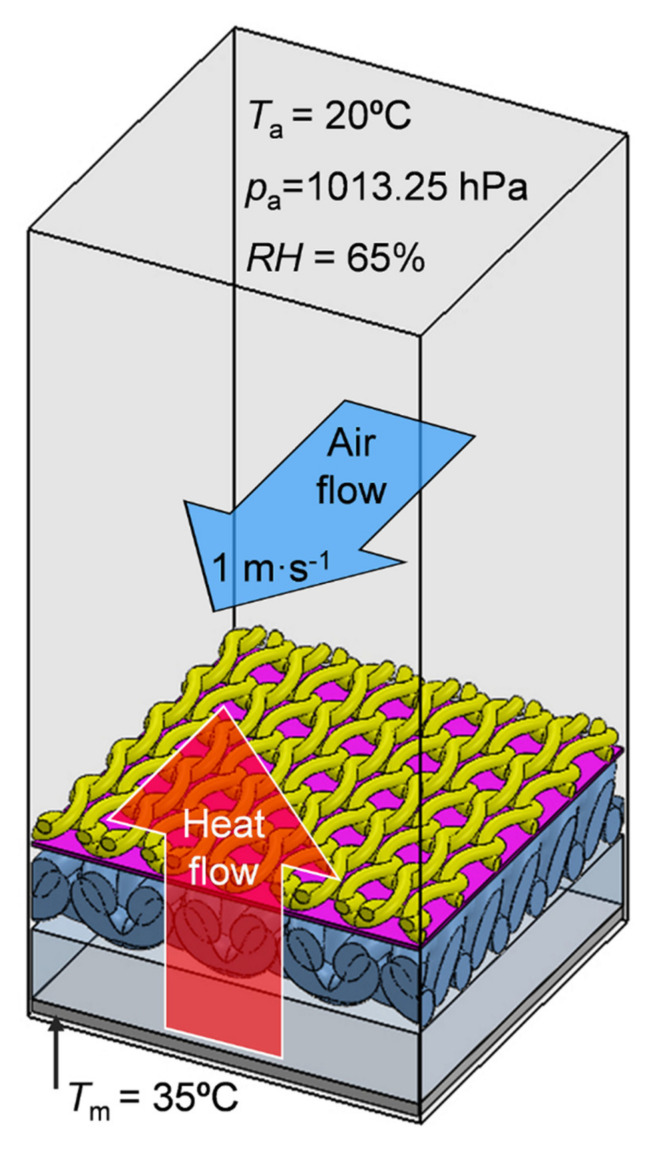
Simulation conditions.

The main aim of the heat transfer simulations was to determine the thermal insulation of six tested assemblies in the same environmental conditions in which the thermal resistance of the real assemblies was measured using the human skin model. For this purpose, each of the six models was placed on a plate model with a constant temperature of 35 °C. The assembly model and plate model were situated inside a rectangular computing domain that was 5 mm high filled with air (Figure 9).

To imitate an infinite 3 layers of assembly propagating outside of computational domain in all four horizontal directions, periodic boundary conditions were applied. The computational domain was divided into three types of cells: solid, gas, and partial. The number of cells was different for each assembly model due to their geometry (Table 2).

Micro-CT 3D reconstructions of real textiles forming the six tested assemblies were very useful in the process of designing textile models. They enabled accurate measurements of the spatial geometry of actual knitted fabrics and vapor-permeable membranes. Moreover, yarn in all three knitted fabrics (made with Cotton LM or Cotton LM or PES) and due to their complex internal structure, both vapor permeable membranes (made with PU or PP) were designed as homogenized three-dimensional objects with physical features (density, specific heat, thermal conductivity) resulting from the corresponding porosity calculated using micro-CT, shown in Table 1. Additionally, in the case of the brushed knitted fabric made using the PES structure, the brushed layer designed as a cuboid with porosity (83.40%) calculated using micro-CT was taken into account. Table 3 presents the basic physical parameters of the raw materials from which the tested assemblies were made determined under normal climate conditions [23,29,41].

Conditions inside the computational domain were consistent with the initial conditions of the thermal resistance measurements, i.e., *T*_a_ = 20 °C, *p*_a_ = 1013.25 h Pa, *RH* = 40%, *T_model_* = 20 °C, temperature heat plate *T*_m_ = 35 °C, and horizontal air flow speed 1 m·s^−1^. As a result of the temperature difference between the heat plate and the surroundings, a heat transfer occurred through the assembly model in a vertical upward direction. As a result of heat transfer, successive layers of the assembly model heat up, reaching a certain constant temperature after reaching a steady state. As a result of the simulation of heat transfer, the temperature difference between the outer layer and inner layer of each assembly was calculated (temperature drop, *D*_T_ (°C) in steady state.

## 3. Results

**Figure 10:**
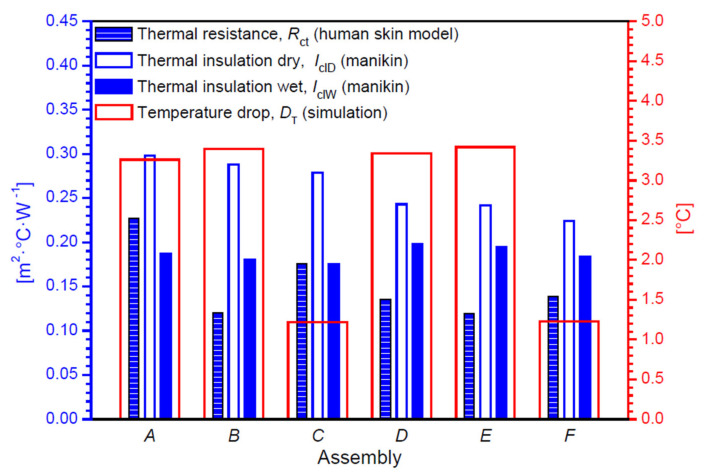
Measured and simulated biophysical properties of tested assemblies.

**Figure 11:**
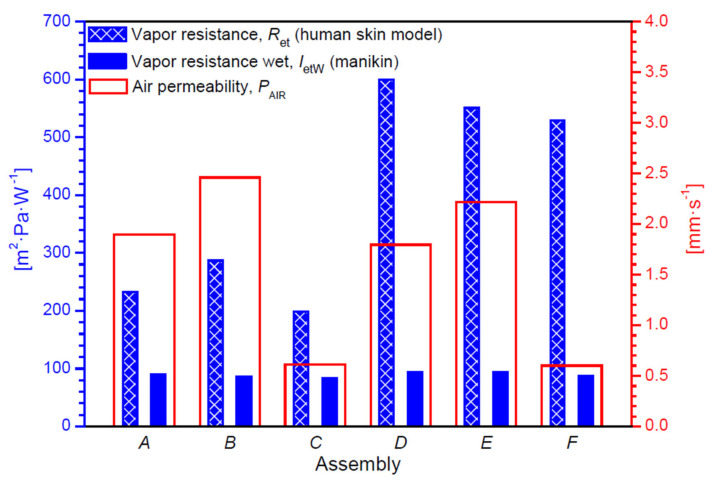
Measured biophysical properties of tested assemblies.

This section presents the results of the research on the biophysical properties and temperature distributions on the 3D models of the six tested assemblies. In Table 4, Figure 10 and Figure 11, the measured and calculated the biophysical properties of the tested textiles are presented.

By analyzing the data presented in Table 4 and in Figure 9, it can be seen that Assembly *A* is characterized by the highest thermal insulation when tested under dry heat flow conditions (0.298 m^2^·°C·W^−1^), and under the conditions in which a sweating manikin was used for testing, the highest thermal insulation performance was achieved by Assembly *D* (0.198 m^2^·°C·W^−1^). Assembly *A* is also characterized by the highest thermal resistance (0.227 m^2^·°C·W^−1^).

An important difference that was observed during the tests is the decrease in the thermal insulation when tested during the simulation of a strong sweating process (3.2·µL·h^−1^·cm^−2^) by about 38% compared to the tests dry heat flow only tests. This means that when a newborn sweats a lot, the drops of sweat formed on the surface of the skin are partially absorbed by the textile material (sleeping bag) and partially evaporated to the outside, which reduces the thermal insulation of the sleeping bag. Nevertheless, the analysis of the obtained results shows that the tested sleeping bags can be used in the following conditions.

Analyzing the data from the table above, it can be seen that the Assemblies *D* and *E* showed the smallest differences in thermal insulation when tested dry and with the use of a sweating manikin. Taking into account the data from Table 4 and Figure 10, it was observed that Assembly *D* had the highest water vapor resistance.

In the case of testing samples using the human skin model, the water vapor resistance of Assembly *D* reached 599.88 m^2^·Pa·W^−1^, and in the case of testing the finished products in the form of a sleeping bag with a thermal manikin, the water vapor resistance was 94.537 m^2^·Pa·W^−1^. The lowest water vapor resistance values are characteristic for Assembly *C* (198.837 m^2^·Pa·W^−1^ using the human skin model and 83.93 m^2^·Pa·W^−1^ using the thermal manikin).

**Figure 12:**
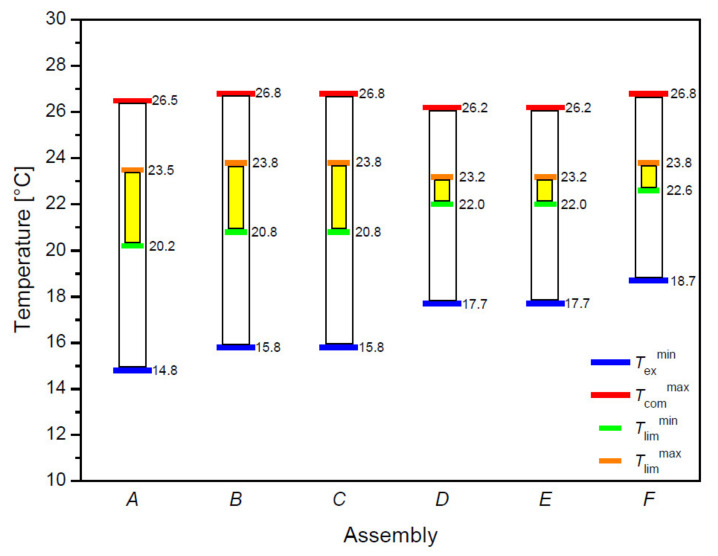
The sleeping bag’s comfort, limit, and extreme temperatures (*T*_com_, *T*_lim_, *T*_ex_) were determined on the basis of the thermal resistance men posture, according to the physiological model described in PN-EN ISO 23537-1:2017-02/A1:2018-05 [42].

The temperature ranges specified in the Table 5 and Figure 12 refer to the results of thermal insulation of the tested systems in the conditions from dry heat flow to the high sweating level of the newborn manikin. The temperature ranges for the three-layer assemblies, which included the PU vapor permeable membrane (Assemblies: *A*–*C*) ranged from 14.8 °C–15.8 °C to 26.5 °C–26.8 °C, while the assemblies with PP vapor permeable membrane (Assemblies *D*–*F*), the ranges were from 17.7 °C–18.7 °C to 26.2 °C–26.8 °C.

**Figure 13:**
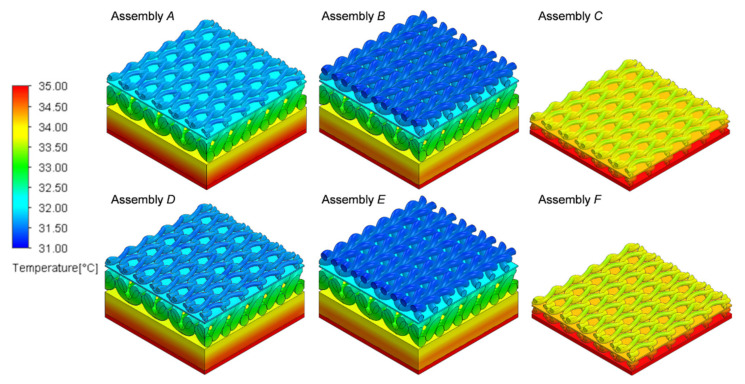
Temperature distributions on 3D models of six tested assemblies.

The results of heat transfer simulations of tested assemblies conducted on 3D models assemblies show the influence of the geometry and the raw material composition of the assembly on its thermal insulation properties. Analyzing the results, it can be concluded that the best heat insulator is the thickest in assemblies *E* and *B,* in which the highest temperature drops *D*_T_ were calculated (Assembly *E*: 3.42 °C and Assembly *B*: 3.40 °C), whereas, the assemblies with the lowest thermal insulation were the thinnest assemblies, *C* and *F,* in which the lowest values of *D*_T_ were calculated (Assembly *C*: 1.22 °C and Assembly *F*: 1.23 °C). Temperature distributions on the 3D models of the tested assemblies are presented in Figure 13.

## 4. Discussion

The presented work was aimed at assessing the biophysical properties of the selected six three-layer assemblies with a different composition of raw materials used in clothing for premature children. The biophysical comfort of a prematurely born child, apart from the sensory features of the clothes worn, is primarily influenced by their biophysical properties. The created clothing systems must act as a heat and moisture insulator so that heat and water are not lost from the body excessively, which would result in the infant becoming hypothermic. Assembly *B* (cotton HM, PU vapor permeable membrane and PES brushed knitted fabric) showed the highest air permeability, with an air permeability value of 2.459 mm·s^−1^, and Assembly *F* (cotton LM, PP vapor permeable membrane, cotton LM) showed the lowest, with an air permeability of 0.601 mm·s^−1^.

The systems with a PU vapor permeable membrane showed higher values of thermal resistance, and the highest result was characterized by Assembly *A* (cotton LM, PU vapor permeable membrane and PES brushed knitted fabric), the value of which was 0.227 m^2^·°C·W^−1^. Thus, the assemblies of these materials best maintain a constant temperature difference between the environment and the child’s skin during the flow of a stream of dry heat. On the other hand, in the case of water vapor resistance, systems containing a PP vapor permeable membrane achieved much higher values than those with a PP vapor permeable membrane. The highest water vapor resistance was shown by Assembly *D* (cotton LM, PP vapor permeable membrane and PES brushed knitted fabric) with a value of 599.882 m^2^·Pa·W^−1^. This arrangement best maintains a constant differential pressure of water vapor on both sides of the garment while heat vapor flows through its surface. The results of the effective thermal insulation in tests with a dry heat flux are higher in clothes from systems containing PU vapor permeable membrane. The highest result of 0.298 m^2^·°C·W^−1^ is shown for Assembly *A* (cotton LM, PU vapor permeable membrane and PES brushed knitted fabric). Therefore, this material is the best heat insulator between the body of a premature baby and its surroundings. In the case of tests for the effective water vapor resistance using a sweating manikin, clothes containing a PP vapor permeable membrane have higher results and the largest difference in water vapor pressure between the infant’s body and the environment, amounting to a 94.537 m^2^·Pa·W^−1^ for a polypropylene vapor permeable membrane and polyester brushed knitted fabric.

Analyzing the results of the heat transport simulation using 3D models of the real assemblies, it can be seen that the temperature difference between the layer of the material near the skin and the outer layer of the garment material is influenced by the thickness of a given system and its raw material composition. The thinnest assemblies containing knitted fabric with cotton LM on both sides of the vapor permeable membrane (Assembly *C* and Assembly *F*) are characterized by the smallest temperature differences (*C*: 1.22 °C, *F*: 1.23 °C). The assemblies containing a PU vapor permeable membrane have slightly higher temperature differences with the same configuration of two identical knitted patterns (e.g., *A*: 3.26 °C and *D*: 3.34 °C). Thus, a PU vapor permeable membrane is a better heat insulator when protecting the baby’s skin from the environment. This is due to the lower thermal conductivity of the PU vapor permeable membrane. The infant’s body has a lower risk of hypothermia by wearing clothes containing PU vapor permeable membrane. On the basis of the results alone, it is difficult to unequivocally conclude which garment is an ideal solution for a prematurely born child because biophysical comfort is influenced by many factors, and some of them are individual for each human body. Following the most important parameters, which are to ensure a sufficiently high temperature and humidity of the child’s skin to prevent premature babies from becoming hypothermic, sleeping bags containing a layer of propylene vapor permeable membrane show higher water vapor resistance (529.43–599.88 m^2^·Pa·W^−1^) than those containing a polyurethane vapor permeable membrane (198.84–286.46 m^2^·Pa·W^−1^). Each of the sleeping bags made from the developed three-layer systems show better biophysical properties, i.e., higher water vapor resistance and thermal insulation than commercially available clothes made of cotton only.

## 5. Conclusions

In this article, the results of the assessment of the biophysical properties of six textile assemblies differing in the raw material composition and geometry used in mummy sleeping bags dedicated to premature infants were presented. The main role of the tested assemblies is to provide the newborn with physiological comfort through optimal heat and mass exchange between its skin and the environment.

The experimental part of the work using real textiles included tests forair permeability, thermal resistance and water vapor resistance (both using human skin model), thermal insulation, and water vapor resistance (both using the newborn manikin).

Additionally, on the basis of the three-dimensional reconstructions obtained using micro-computed tomography, three-dimensional models of the tested real assemblies were designed in order to simulate the thermal insulation of the real textile systems using the finite volume method.

On the basis of the obtained research results, the following conclusions can be drawn:

- In the case of premature newborns, a knitted construction should be used with properties blocking moisture flow and should be characterized by high thermal insulation while ensuring adequate comfort of use. Assembly *D* (0.198 m^2^·°C·W^−1^) showed the best properties. In the case of testing samples using the human skin model, the water vapor resistance of Assembly *D* reached 599.88 m^2^·°C·W^−1^, and in the case of testing the finished products in the form of a sleeping bag with a thermal manikin, the water vapor resistance was 94.537 m^2^·Pa·W^−1^. Moreover, this sample was characterized by good air permeability and a slightly higher surface weight than the other samples. However, it should be remembered that the process of designing clothing for premature babies should keep the area weight as low as possible. This is due to the fact that the skin of a premature baby does not have a stratum corneum structure and should not be loaded down.
- Despite the use of simplifications in the geometry of the three-dimensional models of textiles constituting the six tested assemblies, the results of the performed simulations show the impact of the geometry and the raw material composition of the assembly on its thermal insulation properties. The presented models could be useful in predicting the biophysical comfort of the designed and manufactured textiles containing knitted fabrics and membranes.
- The materials used and the conducted experimental studies supported by numerical modeling constitute a new direction in the field of research on the biophysical comfort of clothing worn by premature infants. These studies can provide valuable information on neonatal care.

## 6. Patents

Krucińska I., Kowalski K., Skrzetuska E., Komisarczyk A. Material for a baby clothing for prematurely born infants, No. Pat. 231994.

## Figures and Tables

**Table 1:** Characteristics of six tested assemblies.

Assembly	Layer nr	Layer Type	Layer Composition	Layer Thickness ^a^ [mm]		Surface Mass ^b^ [g·m^−2^]	Yarn Diameter ^c^ [mm]	Layer Porosity ^d^ [%]	Yarn Porosity ^d^ [%]
***A***	1	Knitted fabric	Cotton LM	0.158	1.636	72.0	0.108	38.64	13.81
	2	Vapor permeable membrane	PU	0.013		14.8	–	2.01	–
	3	Brushed knitted fabric	PES	0.558		106.3	0.246	76.28	30.20
***B***	1	Knitted fabric	Cotton HM	0.310	1.922	104	0.158	26.83	9.41
	2	Vapor permeable membrane	PU	0.013		14.8	–	2.01	–
	3	Brushed knitted fabric	PES	0.558		106	0.246	76.28	30.20
***C***	1	Knitted fabric	Cotton LM	0.158	0.788	72.0	0.108	38.64	13.81
	2	Vapor permeable membrane	PU	0.013		14.8	–	2.01	–
	3	Knitted fabric	Cotton LM	0.158		72.0	0.108	38.64	13.81
***D***	1	Knitted fabric	Cotton LM	0.158	1.656	72.0	0.108	38.64	13.81
	2	Vapor permeable membrane	PP	0.031		34.4	–	1.55	–
	3	Brushed knitted fabric	PES	0.558		106.3	0.246	76.28	30.20
***E***	1	Knitted fabric	Cotton HM	0.310	1.942	104	0.158	26.83	9.41
	2	Vapor permeable membrane	PP	0.031		34.4	–	1.55	–
	3	Brushed knitted fabric	PES	0.558		106	0.246	76.28	30.20
***F***	1	Knitted fabric	Cotton LM	0.158	0.808	72.0	0.108	38.64	13.81
	2	Vapor permeable membrane	PP	0.031		34.4	–	1.55	–
	3	Knitted fabric	Cotton LM	0.158		72.0	0.108	38.64	13.81

^a^ Determined according to PN-EN ISO 5084:1999 [30]; ^b^ determined according to PN EN 12127:2000 [31]; ^c^ determined according to [32]; ^d^ determined using X-ray micro-computed tomography.

**Table 2:** Number of cells in 3D models of tested assemblies.

Assembly	Solid Cells	Gas Cells	Partial Cells
*A*	67,670	66,530	43,520
*B*	71,451	71,873	51,968
*C*	35,034	59,181	29,408
*D*	68,430	67,625	43,782
*E*	72,570	72,980	53,010
*F*	37,127	60,237	30,569

**Table 3:** Physical features of raw materials applied in simulations [23,29,41].

Physical Parameter	Cotton	PES	PU	PP	Air
density [kg·m^−3^]	1550	1370	1260	940	1.2
specific heat [J·kg^−1^·°C^−1^]	1330	1380	1120	1720	1005
thermal conductivity [W·m^−1^·°C^−1^]	0.07	0.08	0.23	0.26	0.03

**Table 4:** Measured and simulated biophysical properties of tested assemblies [23,29].

Assembly	Air Permeability Tester	Human Skin Model		Newborn Manikin			Simulations
	Air Permeability, *P*_AIR_ [mm·s^−1^]	Thermal Resistance, *R*_ct_ [m^2^·°C·W^−1^]	Vapor Resistance *R*_et_ [m^2^·Pa·W^−1^]	Thermal Insulation Dry, *I*_clD_ [m^2^·°C·W^−^^1^]	Thermal Insulation Wet, *I*_clW_ [m^2^·°C·W^−1^]	Vapor Resistance Wet, *I*_etW_ [m^2^·Pa·W^−1^]	Temperature Drop, *D*_T_ [°C]
*A*	1.893	0.227	232.740	0.298	0.187	90.060	3.26
*B*	2.459	0.120	286.459	0.288	0.180	86.268	3.40
*C*	0.613	0.176	198.837	0.279	0.175	83.932	1.22
*D*	1.796	0.135	599.882	0.243	0.198	94.537	3.34
*E*	2.216	0.119	550.409	0.242	0.195	93.221	3.42
*F*	0.601	0.139	529.431	0.224	0.184	87.434	1.23

**Table 5:** The sleeping bag’s comfort, limit, and extreme temperatures (*T*_com_, *T*_lim_, *T*_ex_) were determined on the basis of the thermal resistance mean posture, according to the physiological model described in PN-EN ISO 23537-1:2017-02/A1:2018-05 [42].

Assembly	Thermal Insulation Dry, *I*_clD_ [m^2^·°C·W^−1^]	Thermal Insulation Wet, *I*_clW_ [m^2^·°C·W^−1^]	Extreme Temperature, *T*_ex_ [°C]	Limit Temperature, *T*_lim_ [°C]	Comfort Temperature, *T*_com_ [°C]
*A*	0.298	0.187	14.8–20.2	20.2–23.5	23.2–26.5
*B*	0.288	0.180	15.8–20.7	20.8–23.8	23.8–26.8
*C*	0.279	0.175	15.8–20.7	20.8–23.8	23.8–26.8
*D*	0.243	0.198	17.7–19.7	22.0–23.2	25.0–26.2
*E*	0.242	0.195	17.7–19.7	22.0–23.2	25.0–26.2
*F*	0.224	0.184	18.7–20.7	22.6–23.8	25.6–26.8

## Data Availability

Not applicable.
